# Neuropeptide Y resists excess loss of fat by lipolysis in calorie‐restricted mice: a trait potential for the life‐extending effect of calorie restriction

**DOI:** 10.1111/acel.12558

**Published:** 2017-01-19

**Authors:** Seongjoon Park, Toshimitsu Komatsu, Sang Eun Kim, Katsuya Tanaka, Hiroko Hayashi, Ryoichi Mori, Isao Shimokawa

**Affiliations:** ^1^ Department of PathologyNagasaki University School of MedicineGraduate School of Biomedical Sciences1‐12‐4 SakamotoNagasaki852‐8523Japan; ^2^ Department of Plastic and Reconstructive SurgeryNagasaki University School of MedicineGraduate School of Biomedical Sciences1‐12‐4 SakamotoNagasaki852‐8523Japan

**Keywords:** adipose tissue, aging, calorie restriction, lipolysis, neuropeptide Y

## Abstract

Neuropeptide Y (NPY) is an orexigenic peptide that plays an essential role in caloric restriction (CR)‐mediated lifespan extension. However, the mechanisms underlying the NPY‐mediated effects in CR are poorly defined. Here, we report that NPY deficiency in male mice during CR increases mortality in association with lipodystrophy. NPY ^−/−^ mice displayed a rapid decrease in body weight and fat mass, as well as increased lipolysis during CR. These alterations in fat regulation were inhibited by the lipolysis inhibitor, acipimox, a treatment associated with reduced mortality. The lipolytic/thermogenic signaling, β3‐adrenergic receptor/hormone sensitive lipase, was markedly activated in white adipose tissue of NPY ^−/−^ mice compared with that of NPY ^+/+^ mice, and thermogenesis was controlled by NPY under negative energy balance. These results demonstrate the critical role of NPY in the regulation of lipid metabolic homeostasis and survival via control of lipolysis and thermogenesis in a state of negative energy balance.

## Introduction

Fat, an indispensable component of the cell membrane, also forms a layer under the skin, which provides insulation from both cold and heat. Furthermore, fat is a major energy source in mammals when energy is insufficient (Lafontan & Langin, 2009). However, abnormally high or low amounts of fat can induce obesity or lipodystrophy through altered metabolic homeostasis (Lewington *et al*., 2009; Mao *et al*., 2009). The removal of body fat by surgery promotes lifespan extension (Muzumdar *et al*., 2008), and a low body fat mass is a common phenotype associated with longevity in animal models (Blüher *et al*., 2002; Heiman *et al*., 2003; Koubova & Guarente, 2003), illustrating that fat influences longevity.

Neuropeptide Y (NPY) is a key factor that regulates orexigenic activity and energy homeostasis in the brain (Mercer *et al*., 2011). Fasting stimulates NPY (Sahu *et al*., 1992), which increases food intake and body weight (BW), thereby promoting adiposity when NPY is administered exogenously or overexpressed (Zarjevski *et al*., 1993; Ruohonen *et al*., 2012). Conversely, suppression of NPY signaling decreases BW gain and adiposity in obese mice (Ishihara *et al*., 2006; Chao *et al*., 2011). *In vivo* studies showed that caloric restriction (CR) modifies metabolic patterns, characterized by increased fatty acid synthesis and fatty acid oxidation in adipose tissue, as fatty acids represent an important energy source for metabolic adaptation in CR animals (Bruss *et al*., 2010). We recently found that NPY deficiency disrupts CR‐mediated lifespan extension (Chiba *et al*., 2014). This finding provides the first demonstration that NPY plays an important role in the adaptation to negative energy balance. However, the mechanisms underlying the NPY‐mediated effects of CR are currently unknown.

Lipolysis, the biochemical process that converts triglycerides to glycerol and fatty acids, occurs in response to β‐adrenergic stimulation by catecholamines during fasting and exercise. Lipolysis is regulated by three lipases, adipose triglyceride lipase (ATGL), hormone sensitive lipase (HSL), and monoacylglycerol lipase. Stimulation of β‐adrenergic receptors activates adenylyl cyclase, which triggers activation of cAMP‐dependent protein kinase A (PKA). PKA activates ATGL, comparative gene identification 58 alpha (CGI‐58), and HSL, resulting in the induction of lipolysis (Duncan *et al*., 2007). β3‐adrenergic receptor (Adrb3) is abundantly expressed in rodent adipose tissue (Nahmias *et al*., 1991) and promotes the remodeling of white adipose tissue (WAT) by stimulating cell proliferation and catabolic capacity (Granneman *et al*., 2005). In addition, Adrb3 agonists have been extensively studied in rodents as potential antidiabetes and anti‐obesity agents (Arch & Wilson, 1996; Ghorbani *et al*., 2012). Although moderate lipolysis is essential role for regulating lipid turn over and adiposity, excessive lipolysis is also involved in disease states such as cancer and cachexia (Arner & Langin, 2014).

To better understand the functional role of NPY in adipose tissue metabolism under negative energy balance, we performed CR in NPY‐disrupted mice. We observed that NPY deficiency enhanced lipolysis associated with the abnormal activation of Adrb3‐HSL signaling, resulting in the induction of excessive fat loss and promotion of increased mortality during CR. However, ACM treatment inhibited excessive lipolysis and decreased mortality. Thus, our data demonstrate that NPY is essential for survival during negative energy balance by promoting the maintenance of lipid metabolic balance via lipolytic regulation.

## Results

### NPY deficiency increases mortality in CR mice, which is inhibited by ACM treatment

**Figure 1:**
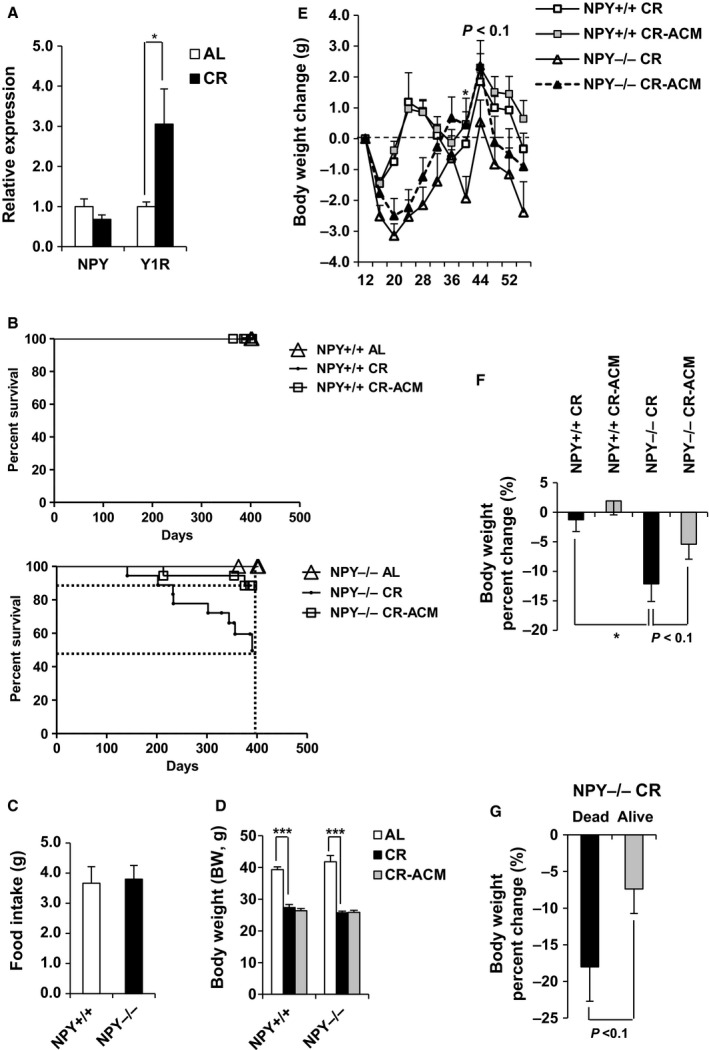
NPY deficiency increases mortality with reduction in body weight in CR mice, which is inhibited by ACM treatment. (A) mRNA expression level of *Y1R* was measured by qPCR in WAT. (B) Survival rate was measured by the log‐rank test (*n* = 18, upper panel). The dotted line indicates the percent survival at 400 days. (C) Food intake, (D) body weight, and (E–G) change of body weight were measured in NPY ^+/+^ and NPY ^−/−^ mice. All data are presented as the mean ± SEM,* n* = 12–18 per group; *P < 0.05 and ***P < 0.001.

The recently reported involvement of NPY in adipose tissue metabolism and lifespan extension during CR in mice (Chiba *et al*., 2014; Park *et al*., 2014) prompted us to investigate the functional role of NPY in CR mice. In a previous study, we found that body fat was markedly reduced and mortality was increased in NPY^−/−^ mice, compared with that in NPY^+/+^ mice during CR (Chiba *et al*., 2014). To explore whether fat loss promotes increased mortality in NPY^−/−^ mice during 30% CR, we treated NPY^+/+^ and NPY^−/−^ mice with the lipolysis inhibitor, acipimox (ACM), upon CR initiation. CR increased the mRNA expression level of *Npy1r* (Y1R) but not *Npy* in white adipose tissue (WAT) (Fig. 1A). Moreover, we showed that all wild‐type mice survived, whereas 44% of knockout mice died within one year of CR (Fig. 1B). Interestingly, oral ACM treatment during CR decreased mortality in NPY^−/−^ mice, but ACM treatment during CR did not affect mortality in NPY^+/+^ mice (Fig. 1B). At 400 days, only 56% of NPY^−/−^ CR mice were alive compare with 89% of NPY^−/−^ CR‐ACM‐treated counterparts (Fig. 1B), suggesting that NPY deficiency induced fat loss and promoted increased mortality in CR mice.

### NPY^−/−^ mice display reduction in body weight, fat mass, and adipocyte size during CR, which is inhibited by ACM treatment

**Figure 2:**
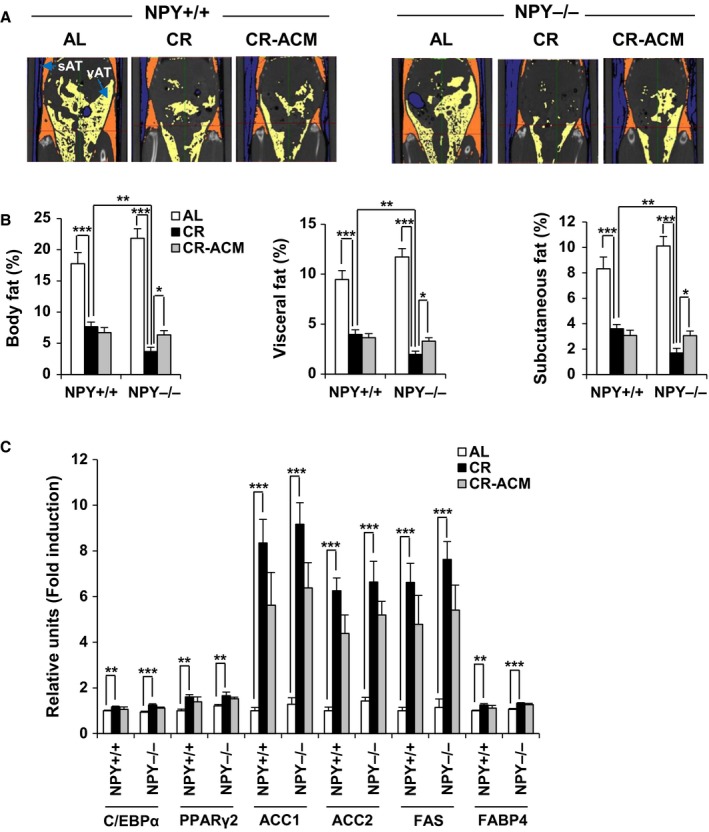
NPY ^−/−^ mice exhibit reduction in fat mass independent of adipogenic regulation during CR, which is inhibited by ACM treatment. (A, B) Percentage of total, visceral, and subcutaneous adipose tissue was determined by 3D micro‐CT. (C) mRNA expression levels of adiponetic and lipogenic genes were measured by qRT–PCR in eWAT. All data are presented as the mean ± SEM,* n* = 9–11 per group; *P < 0.05, **P < 0.01, and ***P < 0.001.

**Figure 3:**
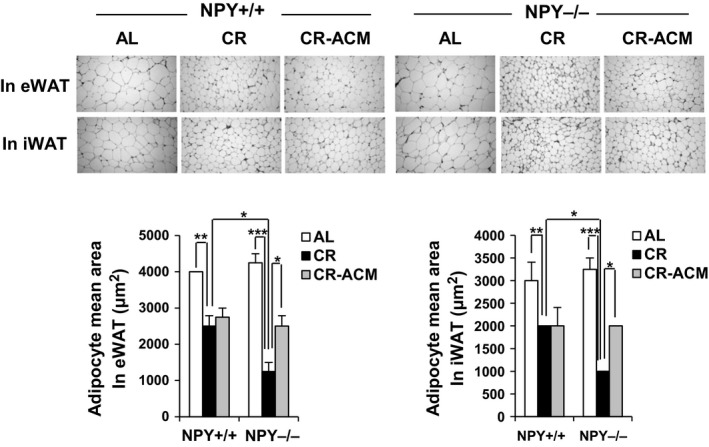
NPY ^−/−^ mice exhibit reduction in adipocyte size during CR, which is inhibited by ACM treatment. Representative images from immunohistochemistry for H&E (hematoxylin and eosin) stain in eWAT and iWAT (upper panel), and the mean sizes of adipocytes (bottom panel). All data are presented as the mean ± SEM,* n* = 6 per group; *P < 0.05, **P < 0.01, and ***P < 0.001.

Food intake was equivalent between mice of either genotype (Fig. 1C), and BW was not different between NPY^+/+^ CR and NPY^−/−^ CR mice when live mice were analyzed (Fig. 1D). However, the BW of all NPY^−/−^ mice during CR (alive and dead) rapidly decreased compared with that of wild‐type mice. For CR only, two‐way (genotype and treatment) ANOVA analyses performed in each strain showed a significant effect of genotype (*P* < 0.001) on BW, but not treatment (*P* = 0.11) or genotype and treatment interaction (*P* = 0.37; Fig. 1F). In particular, dead NPY^−/−^ CR mice displayed a trend of decreased BW compared with that of live NPY^−/−^ CR mice (Fig. 1G). The reduction in BW in NPY^−/−^ CR mice was marginally suppressed by ACM treatment (Fig. 1E,F). Total body fat mass (visceral and subcutaneous fat), as determined using micro‐CT, was significantly reduced by CR (Fig. 2A,B). Two‐way (genotype and diet) ANOVA analyses performed in each strain showed a significant effect of diet (*P* < 0.001) and genotype and diet interaction (*P* < 0.01), but not genotype (*P* = 0.41). Body fat mass was reduced by CR in both NPY^−/−^ and NPY^+/+^ mice, but it was further reduced in NPY^−/−^ CR mice, compared with that in NPY^+/+^ CR mice. ACM treatment inhibited excessive fat loss in NPY^−/−^ CR mice (Fig. 2A,B). We next analyzed multilocular/small lipid morphology, characteristic of brown fat cells in WAT. CR increased these morphological changes in the WAT of both NPY^−/−^ and NPY^+/+^ mice (Fig. 3). Interestingly, these were further increased in the WAT of NPY^−/−^ mice, compared with that of NPY^+/+^ mice, and it was restored by ACM treatment. However, these morphological changes were not induced by NPY deficiency under *ad libitum* feeding conditions (Fig. 3). These results indicate that NPY deficiency induces white fat remodeling under CR feeding condition.

### NPY deficiency enhances lipolysis through activation of HSL in WAT of CR mice, which is inhibited by ACM treatment

**Figure 4:**
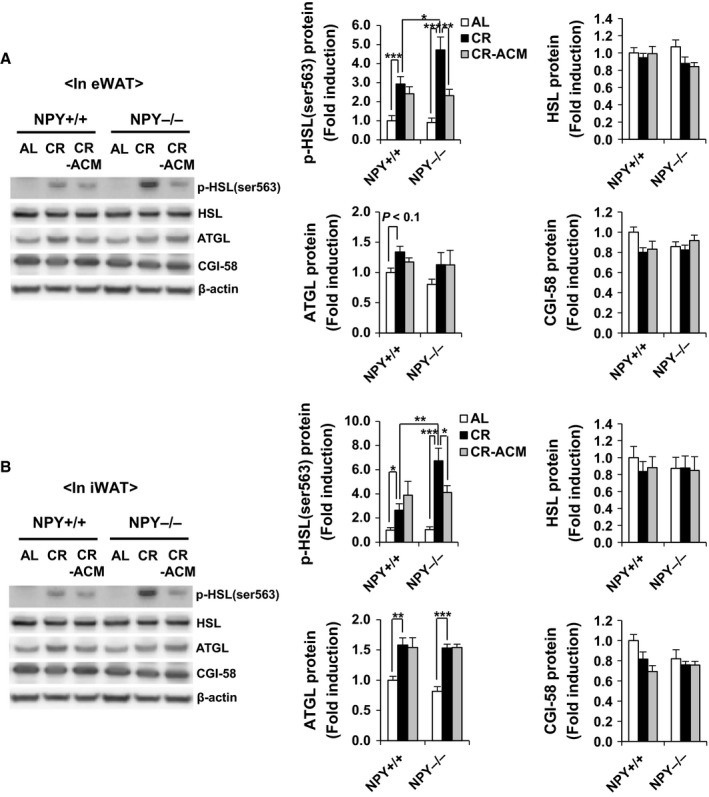
NPY deficiency increases p‐HSL (ser563), but it is inhibited by ACM in WAT of CR mice. Protein level of p‐HSL (ser563), HSL, ATGL, and CGI‐58 was measured by Western blotting in (A) eWAT and (B) iWAT of same mice (left panel) and quantified (right panel). All data are presented as the mean ± SEM;* n* = 6 per group. *P < 0.05, **P < 0.01, and ***P < 0.001.

We reported that NPY promotes adipogenesis and lipid accumulation but inhibits lipolysis in mice (Park *et al*., 2014). To investigate whether NPY deficiency in CR mice directly influences adipogenesis, we compared mRNA expression of adipogenic and lipogenic genes, including those encoding CCAAT/enhancer binding protein alpha (*C/EBP*α), peroxisome proliferator‐activated receptor gamma 2 (*PPAR*γ*2*), acetyl‐CoA‐carboxylase (*ACC*) *1*,* ACC2*, and fatty acid synthase (*FAS*) in epididymal WAT (eWAT) from NPY^+/+^ CR and NPY^−/−^ CR mice. However, no differences were observed between genotypes, although CR enhanced mRNA expression of these genes (Fig. 2C). Activation of the cyclic AMP (cAMP)/protein kinase A (PKA) pathway regulates expression of the lipolytic protein, HSL, thereby stimulating lipolysis. As we previously reported that NPY is involved in regulating the active form of HSL, phosphorylated (p)‐HSL at Ser563 (Park *et al*., 2014), we analyzed this active form of the enzyme. The protein level of p‐HSL (Ser563) was significantly higher in eWAT and inguinal WAT (iWAT) of NPY^−/−^ CR mice than in those of NPY^+/+^ CR mice, and these increases were suppressed by ACM treatment (Fig. 4A,B). The protein level of p‐HSL (Ser563) was not modified by ACM treatment in both eWAT and iWAT of NPY^+/+^ CR mice (Fig. 4A,B). Protein levels of the triglyceride lipase, ATGL, and its co‐lipase, CGI‐58, were not modified by genotype or ACM treatment although the protein level of ATGL was increased by CR (Fig. 4A,B). These results indicate that NPY deficiency reduces body fat mass during CR through activation of HSL‐mediated lipolysis.

### NPY deficiency enhances white fat remodeling through Adrb3‐signaling in WAT of CR mice, which is inhibited by ACM treatment

**Figure 5:**
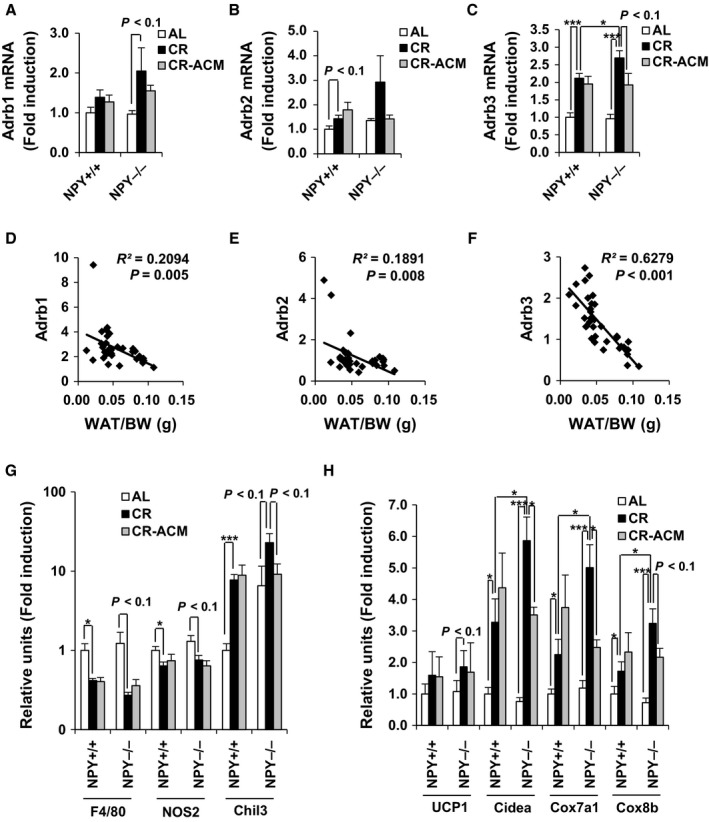
NPY deficiency increases *Adrb3* expression, but it is moderately inhibited by ACM in WAT of CR mice. (A–C) mRNA expression level of *Adrb1, 2, 3* in iWAT of same mice. (D–F) Correlation of *Adrb1, 2, 3* mRNA levels in iWAT with WAT/BW ratio. mRNA expression of genes encoding (G) M1, M2 macrophage markers and (H) thermogenesis was measured by qRT–PCR in iWAT. All data are presented as the mean ± SEM;* n* = 5–6 per group. *P < 0.05 and ***P < 0.001.

Activation of the β‐adrenergic receptor in WAT leads to increased lipolysis and white fat remodeling (Lee *et al*., 2013), and it activates HSL (Mottillo *et al*., 2007). We found that expression of genes encoding β‐adrenergic receptors was increased in iWAT of NPY^−/−^ CR mice, compared with that of NPY^+/+^ CR mice based on results of next‐generation sequencing (data not shown). We confirmed this finding using quantitative real‐time PCR and found that *Adrbs* (β‐adrenergic receptors) expression and body fat mass were negatively correlated (Fig. 5D–F). Furthermore, mRNA expression of the gene encoding the *Adrb3* was significantly increased in iWAT of NPY^−/−^ CR mice, compared with that of NPY^+/+^ CR mice, and ACM treatment inhibited this increase (Fig. 5C). However, the expression levels of genes encoding the *Adrb1,2* were not significantly modified by CR or ACM treatment (Fig. 5A,B).

To test whether induction of Adrb3 signaling highly enhances white fat remodeling in the WAT of NPY^−/−^ mice, compared with that of NPY^+/+^ mice, we further explored multilocular/small lipid morphology characteristic of brown fat cells in iWAT, following an injection of Adrb3 agonist, CL‐316,243 (CL). CL reduced body weight and increased multilocular/small lipid morphology in both NPY^+/+^ and NPY^−/−^ mice; however, these changes were especially more potent in iWAT of NPY^−/−^ mice, compared with those in the iWAT of NPY^+/+^ mice (Fig. S4). These *in vivo* results indicate that lipolysis by induction of Adrb3 signaling is more potent in NPY^−/−^ mice compare with NPY^+/+^ mice. Because recent studies have shown that Adrb3 activation‐induced white fat remodeling involved the death of white adipocytes and their removal by M2 macrophages (Lee *et al*., 2013), we examined the mRNA expression of M1 and M2 macrophage markers, *F4/80* (pan), *NOS2* (M1), and chitinase‐like 3 (*Chil3*, M2) in iWAT. CR reduced the expression of *F4/80* and *NOS2* but increased *Chil3* expression in iWAT of both NPY^+/+^ and NPY^−/−^ mice (Fig. 5G). Furthermore, the expression of *Chil3* was further increased by NPY deficiency during CR. ACM treatment did not affect the expression of these genes in iWAT of NPY^−/−^ AL mice, but significantly inhibited *Chil3* expression in the iWAT of NPY^−/−^ CR mice (Fig. 5G). These results demonstrated that NPY deficiency enhances white fat remodeling through Adrb3‐HSL signaling‐mediated lipolysis and M2 macrophage infiltration under CR feeding condition.

### NPY controls thermogenesis under the negative energy balance

**Figure 6:**
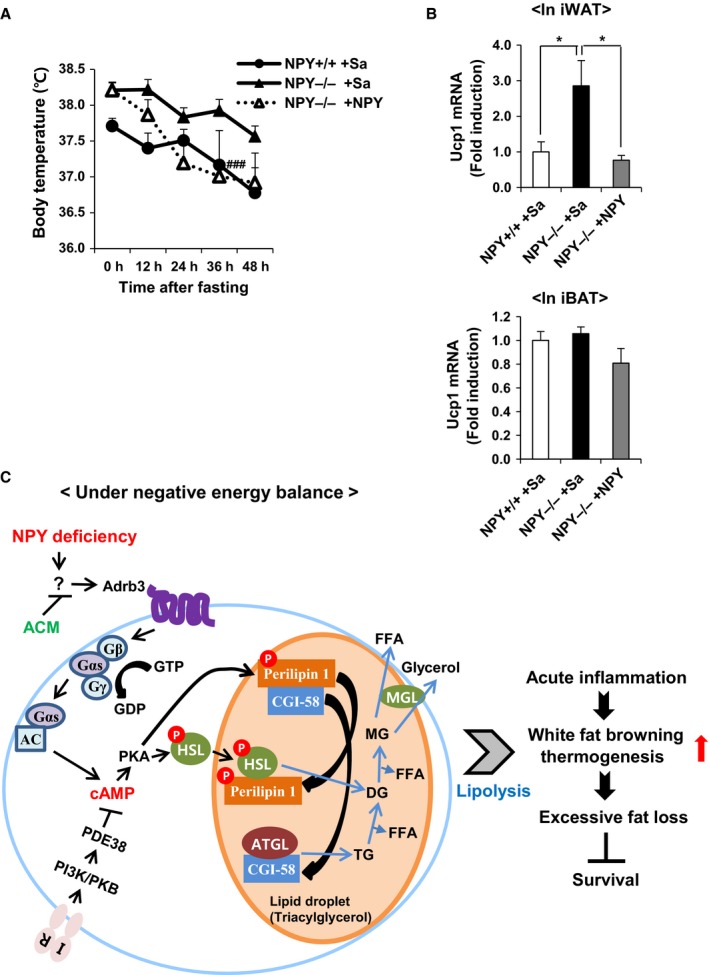
Administration of NPY reduces thermogenesis in NPY ^−/−^ mice under fasted status. (A) Body temperature (B) mRNA expression level of *Ucp1* in iWAT and iBAT. (C) NPY deficiency enhances lipolytic action through activation of Adrb3‐HSL signaling pathway, and ACM inhibits them under negative energy balance. NPY deficiency increases mortality accompanied by BW reduction and fat loss, and it may be due to the increase in lipolysis‐mediated thermogenic action. All data are presented as the mean ± SEM;* n* = 6 per group. *P < 0.05; ^###^ *P* < 0.001 vs. NPY ^−/−^ +Sa.

Increased thermogenesis promotes cachexia (Petruzzelli *et al*., 2014). To test whether NPY controls thermogenesis in CR mice, we measured mRNA expression of genes involved in thermogenesis in iWAT. Two‐way (genotype and diet) ANOVA performed for analyzing the expression of *Cidea, Cox7a1,* and *Cox8b* (Fig. 5H) showed a significant effect of diet (*P* < 0.001), genotype (*P* < 0.01), and genotype and diet interaction (*P* < 0.05). Furthermore, the expression of these thermogenic genes was significantly increased in iWAT of NPY^−/−^ mice, compared with that of NPY^+/+^ mice during CR, and it is reversed by ACM treatment. However, *Ucp1* expression was slightly increased by only CR, per the two‐way (genotype and diet) ANOVA analysis (diet, *P* < 0.01; Fig. 5H). We hypothesized that thermogenesis might be controlled by NPY under the negative energy balance. To address this hypothesis, we analyzed body temperature as well as mRNA expression of *Ucp1* in iWAT, following administration of NPY in fasted‐NPY^−/−^ mice. Body temperature and *Ucp1* expression were significantly reduced by NPY administration, but mRNA expression of *Ucp1* in interscapular brown adipose tissue (iBAT) was not altered (Fig. 6A,B). These results support the notion that NPY suppresses thermogenesis in iWAT in a state of negative energy balance.

## Discussion

Investigating the mechanisms of longevity based on the metabolic characteristics of CR is a promising approach to understand the primary factors responsible for lifespan extension. There are many genes involved in lifespan extension by CR, including those that encode, GH‐IGF1, SIRT1, FOXO3, and adiponectin (Guarente & Picard, 2005; Katic & Kahn, 2005; Otabe *et al*., 2007; Shimokawa *et al*., 2015). Our previous study showed that NPY is also implicated, as NPY deficiency inhibited CR‐mediated lifespan extension (Chiba *et al*., 2014). However, the functional role of NPY in CR remains unclear.

Fat contains 2.25 times the caloric content per gram of proteins and carbohydrates. Thus, fat is a useful energy source for CR animals. Suppression of lipogenesis induces metabolic diseases and early death in mice (Moitra *et al*., 1998; Mao *et al*., 2009), indicating that a proper amount of body fat is essential for life. In addition, the finding that mice with a higher fat content live longer (Liao *et al*., 2011) provides evidence that fat plays an important role in lifespan regulation. Although NPY antagonism was shown to be beneficial under positive energy balance (Ishihara *et al*., 2006; Chao *et al*., 2011), NPY appears to be necessary under negative energy balance. Here, we showed that NPY deficiency reduces BW and WAT mass and increases mortality in CR mice. These results suggest that NPY is essential for metabolic adaptation, which is mediated by maintenance of lipid metabolic balance under negative energy balance.

The present experiment did not directly test the effect of ACM on overall lifespan in NPY^−/−^ CR mice. However, Liao *et al*. clearly indicated a correlation between body fat maintenance and the life‐extending effect of CR, that is, strains of mice displaying a smaller reduction in body fat with 40% CR showed a life‐extending effect of CR, whereas mice a higher reduction in body fat did not show an extended lifespan. Other studies also reported that low body fat content resulting from imbalance of fat metabolism induces metabolic diseases and early death in mice (Moitra *et al*., 1998; Mao *et al*., 2009), indicating that a proper amount of body fat is essential for life. Because lipolysis is activated in CR mice during the fasting phase of the feeding cycle, a mechanism that prevents excess loss of fat contents by lipolysis should underlie the effect of CR. In the present study, the body weight and fat content were extremely lower in NPY^−/−^ CR mice than in NPY^+/+^ CR mice. In dead mice in the NPY^−/−^ CR group, the body weight was significantly lower than that in live NPY^−/−^ CR mice, suggesting an excessive loss of body fat content prior to death. The present study indicates that ACM ameliorates the excessive loss of fat through inhibition of lipolytic action during CR in NPY^−/−^ mice. This suggests involvement of NPY in a trait potential for the life‐extending effect of CR. We previously reported that NPY deficiency did not affect survival during the acute phase of CR in female mice, but decreased the survival of male mice (Chiba *et al*., 2014). Body weight was not significantly changed during CR in female NPY^−/−^ mice, compared with that in NPY^+/+^ mice, although it was decreased by NPY deficiency during CR in male mice (Chiba *et al*., 2014). This indicates that NPY deficiency may affect energy metabolism sex‐dependently. NPY and estrogen are negatively correlated (Dhillon & Belsham, 2011), and estrogen is involved in energy metabolism in female mice (Brown & Clegg, 2010), suggesting that estrogen may facilitate metabolic adaptation during CR through modulation of energy metabolism in female mice. Further studies are essential to verify the correlation between estrogen and NPY.

Several lines of evidence support the concept that NPY is important for cardiac function (Michalkiewicz *et al*., 2003; Dvorakova *et al*., 2014). In addition, lipodystrophy is closely associated with heart failure (Nelson *et al*., 2013). To examine whether the early death of NPY^−/−^ CR mice was promoted by heart failure, we evaluated mRNA levels of cardiac injury and stress genes in heart tissue of NPY^+/+^ CR and NPY^−/−^ CR mice. However, there were no differences in gene expression between the two groups (Fig. S1).

Losses of BW and fat mass predominantly occur during aging and in disease states, such as cachexia and lipodystrophic disorders. Furthermore, these are associated with a doubling in mortality risk (Morley, 2007). NPY is an orexigenic peptide, and anorexia is one of the causes of weight loss. However, anorexia is unlikely to cause increased mortality in NPY^−/−^ CR mice, as food intake was not different in NPY^−/−^ mice, compared with that in NPY^+/+^ mice (Fig. 1C), and all food was completely consumed by both strains. Decreases in NPY levels and nitric oxide activity are major causes of anorexia of aging (Gruenewald *et al*.,^1994^; Morley *et al*., 1996), suggesting that NPY could play a key role in preventing anorexia of aging. Our results showed that mRNA expression of *Adrb3* and HSL activity was increased in WAT of NPY^−/−^ CR mice, compared with those of NPY^+/+^ CR mice (Figs 4 and 5). Therefore, excessive lipolysis could promote weight and fat loss in NPY^−/−^ CR mice. The findings that ACM treatment suppresses fat and weight loss and decreases mortality associated with NPY deficiency under CR demonstrate that excessive lipolysis by NPY deficiency is associated with increased mortality.

Stimulation of *Adrb3* expression promotes WAT remodeling concomitant with transient inflammation, increased mitochondrial biogenesis, and increased fatty acid oxidation. Analysis of cellular plasticity indicated that HSL plays an important role in tissue remodeling (Mottillo *et al*., 2007). Thus, the reduction in body fat mass and adipocyte size in WAT of NPY^−/−^ CR mice could be due to cellular adaptation to the excessive efflux of fatty acids induced by activation of the Adrb3‐HSL signaling pathway. In addition, the ratio of WAT/BW was negatively correlated with expression of *Adrbs* and that of various inflammatory markers, including *Ccl2*,* Ccl7*, and *Il‐6* (Figs 5 and S2), especially in the WAT of NPY^−/−^ CR mice. These findings indicate that β‐adrenergic remodeling was strongly induced. The results from *in vitro* experiments showed that treatment with NPY or ACM reduced isoproterenol‐induced free fatty acid release in 3T3‐L1 adipocytes (Fig. S3). These *in vivo* and *in vitro* results suggest that the lipolytic action and associated WAT remodeling are regulated by NPY.

Cachexia is a common problem in cancer but also in nonmalignant states, such as chronic infection, heart and renal failure, as well as chronic inflammatory diseases (Morley *et al*., 2006). Survival of cancer patients can decrease at 5% weight loss (Dewys *et al*., 1980). In addition, weight loss approaching 30% in incompatible with life (Fearon, 1992), which suggests that prevention and treatment of cachexia are essential. Metabolic dysfunction and an increased metabolic rate could be major causes of cancer‐associated cachexia. Systemic inflammation, weight loss, and WAT atrophy are predominantly observed in cancer cachexia (Blum *et al*., 2011). It has been proposed that reduction in white fat browning by Adrbs inhibition and anti‐inflammatory treatment ameliorates the severity of cachexia (Petruzzelli *et al*., 2014). Here, we showed that NPY deficiency significantly increased expression of *Adrb3* in WAT (Fig. 5C) concomitant with reductions in BW and fat mass during negative energy balance. In addition, expression of inflammatory cytokines was negatively correlated with fat mass, especially in NPY^−/−^ CR mice (Fig. S2). The activation of Adrb3‐HSL signaling promotes white fat browning, and it is associated with increased thermogenesis, resulting in increased lipid mobilization and energy expenditure (Petruzzelli *et al*., 2014). Consistent with the expression pattern of other thermogenic genes, *Ucp1* expression was not significantly increased by CR or NPY deficiency. Adrb3 activation of Ucp1^−/−^ mice increases metabolic rate in WAT (Granneman *et al*., 2003), suggesting that upregulation of Adrb3 signaling by NPY deficiency induces thermogenesis by UCP1 independently. Further studies are warranted to verify the UCP1‐independent mechanisms by NPY deficiency during CR. Energy expenditure per unit body mass did not differ, particularly between male NPY^−/−^ CR and NPY^+/+^ CR mice; however, this was measured at the age of 7 months, in a previous study (Chiba *et al*., 2014). We expect that further study of the energy expenditure after the age of 7‐months, when NPY^−/−^ CR mice gradually died, might be useful for better understanding.

In summary, our study demonstrates the critical role of NPY in the regulation of fat metabolism under negative energy balance. NPY deficiency increased mortality in CR mice in association with BW reduction and fat loss by upregulating lipolysis/thermogenesis via abnormal activation of the Adrb3‐HSL signaling pathway, whereas ACM treatment was inhibitory. Furthermore, NPY controlled thermogenesis in NPY^−/−^ mice under negative energy balance (Fig. 6C). Our study using NPY^−/−^ mice with negative energy balance supports a mechanism consisting of NPY control of the Adrb3‐HSL pathway and shows that maintenance of fat metabolism by NPY is a potential therapeutic strategy for prevention of frailty in elderly people those with various diseases such as cancer.

## Experimental procedures

### Animals

The animal care and experimental protocols were approved by the Ethics Review Committee for Animal Experimentation at Nagasaki University. Male NPY^−/−^ mice (129S‐Npytm1Rpa/J) and female NPY^+/+^ mice (129S6/SvEvTac) were obtained from Jackson Laboratory (Bar Harbor, ME, USA) and Taconic Farms, Inc. (Germantown, NY, USA). They were bred in a barrier facility at the Center for Frontier Life Sciences at Nagasaki University. NPY‐null allele maintained on a mixed genetic background derived from intercrosses between the NPY^+/+^ mice (129S6/SvEvTac) and NPY^−/−^ mice (129S‐Npytm1Rpa/J). Three mice were housed in individual cages in the barrier facility (temperature, 21–24 °C; 12 h light/dark cycle) under specific pathogen‐free conditions, which were maintained for the entire study. All mice were fed *ad libitum* (AL) with Charles River Formula 1 (CRF‐1) diet (Oriental Yeast Co. Ltd., Tokyo, Japan). At 12 weeks of age, mice were divided into AL and CR groups. The CR groups received a food allotment consisting of 70% of the mean daily food intake of the AL groups of male NPY^−/−^ and WT mice every day, 30 min before lights were turned off. The food allotments for the CR groups were adjusted every 2 weeks between 12 and 32 weeks; the allotments were fixed between 32 and 56 weeks. Details of the feeding procedure have been previously described (Yamaza *et al*., 2010). Acipimox (LKT Laboratories, Inc., St. Paul, USA) was provided in drinking water (at a concentration of 0.05%) and replaced twice a week. Body weight was monitored every 2 weeks between 6 and 12 weeks of age and every 4 weeks thereafter. At the 12 months old, percentage of body fat was measured using 3D micro‐CT (Rigaku Co., Tokyo, Japan). For NPY treatment, mice were fasted and injected intraperitoneally (i.p.) with 120 μg kg^−1^ NPY (Tocris Bioscience, Minneapolis, USA) in saline every 24 h for 2 days and sacrificed 3 h after last injection.

After serum samples collected from cardiac puncture, mice were sacrificed and tissues were immediately collected.

### Western blotting

Antibodies for p‐HSL (ser563), HSL, and ATGL were obtained from Cell Signaling Technology (Danvers, MA, USA). Anti‐β‐actin was purchased from Abcam (Cambridge, UK), and anti‐CGI58 was obtained from Santa Cruz Biotechnology (Santa Cruz, CA, USA). Enhanced chemiluminescence (ECL) Western blotting detection reagents and ECL‐anti‐rabbit or mouse IgG, horseradish peroxidase‐linked species‐specific whole antibodies, were purchased from Amersham Pharmacia Biotech (Little Chalfont, UK).

All samples were boiled for 3 min and chilled on ice. Proteins were separated by SDS‐PAGE and transferred to a nitrocellulose membrane. Membrane was immediately placed in blocking solution (3% BSA or 5% nonfat dry milk in TBS‐T buffer) for 1 h and incubated with the primary antibody for overnight at 4 °C followed by the secondly antibody for 1 h at room temperature. Antibody labeling was detected using ECL as per the manufacturer's instructions. Specific signals were quantified by Fluorchem (DE500‐5T; Alpha Innotech Corporation, San Leandro, CA, USA) with associated image analysis software, AlphaEase FC.

### Quantitative real‐time PCR

Total RNA was isolated using RNeasy tissue kit (Qiagen, Valencia, USA), and cDNA was synthesized using ReverTra qPCR RT kit (Toyobo, Osaka, Japan). The relative amount of mRNA expression was analyzed by quantitative PCR (qPCR) with THUNDERBIRD™ SYBR qPCR Mix (Toyobo) according to the protocol provided by the manufacturer. The results were normalized by the 18S expression levels. See Table 1 for primer sequences.

**Table 1:** Gene name, symbols, and primer sequences

Gene name	Gene symbol	Forward primer	Reverse primer
18s ribosomal RNA	18S	TTCTGGCCAACGGTCTAGACAAC	CCAGTGGTCTTGGTGTGCTGA
Acetyl‐Coenzyme A carboxylase 1	Acc1	TGGCAGACCACTATGTTCCA	GTTCTGGGAGTTTCGGGTTC
Acetyl‐Coenzyme A carboxylase 2	Acc2	CCTGTTGCCCAAGAGAGAG	ACAGCGGTCAGGTCAAAGTT
Acyl‐CoA Thioesterase 1	Acot1	ACCCCGAGGTAAAAGGACCT	TTGCAAAGCATCTACAACATCC
β‐adrenergic receptor 1	Adrb1	GTAACGTGCTGGTGATCGTG	AAGTCCAGAGCTCGCAGAAG
β‐adrenergic receptor 2	Adrb2	GAGCACAAAGCCCTCAAGAC	GTTGACGTAGCCCAACCAGT
β‐adrenergic receptor 3	Adrb3		
Chemokine ligand 2	Ccl‐2	TTAAAAACCTGGATCGGAACCAA	GCATTAGCTTCAGATTTACGGGT
Chemokine ligand 7	Ccl‐7	GCTGCTTTCAGCATCCAAGTG	CCAGGGACACCGACTACTG
CCAAT enhancer binding protein alpha	C/EBPα	TTGAAGCACAATCGATCCATCC	GCACACTGCCATTGCACAAG
Fatty acid synthase	Fas	AAGGCTGGGCTCTATGGATT	GGAGTGAGGCTGGGTTGATA
Interleukin 6	Il‐6	TAGTCCTTCCTACCCCAATTTCC	TTGGTCCTTAGCCACTCCTTC
Myosin heavy chain 6	Myh6	GCAGCTGTGCATCAACTTCAC	CACTCAATGCCCTCCTTCTTG
Myosin heavy chain 7	Myh7	TGAATGAGCACCGGAGCAA	CTGGCTGGTGAGGTCATTGA
Natriuretic peptide A	Nppa	TCGTCTTGGCCTTTTGGCT	TCCAGGTGGTCTAGCAGGTTCT
Neuropeptide Y	Npy	CTGCGACACTACATCAATCTCATCA	CAGTGTCTCAGGGCTGGATCTC
Peroxisome proliferator‐activated receptor gamma 2	Pparγ2	TGGGTGAAACTCTGGGAGAT	GCTGGAGAAATCAACTGTGG
Uncoupling protein 1	Ucp1	CACTCAGGATTGGCCTCTACGAC	GCTCTGGGCTTGCATTCTGAC
Neuropeptide Y1 receptor	Y1R	GTCCTTGCAGTGGCTTCTTC	TGATTCGCTTGGTCTCACTG

### Histological analyses

Adipose tissue was fixed in 4% paraformaldehyde (PFA), embedded in paraffin, and stained with hematoxylin and eosin (H&E) staining.

### Body temperature

The body temperature of mice was measured with rectal thermometers (TERUMO CTM‐303; Terumo Corporation, Tokyo, Japan). The rectal probe (ME‐PDK061; Terumo Corporation) was lubricated with glycerol and then inserted into the rectum about 3 cm. The rectal temperature measurement procedure took one minute.

### Statistical analysis

All data are presented as means ± SEM and were analyzed by unpaired two‐tailed Student's *t*‐test and two‐way ANOVA test. Statistical analysis was performed with GraphPad Prism 5, and *P *< 0.05 was considered significant.

## Author contributions

S.P. conducted all experiments and wrote the manuscript. T.K. helped analysis of 3D micro‐CT and CR experiments. S.E.K, K.T., H.H., and R.M. served as advisors for histological analysis and animal care. I.S. helped experimental design, data analysis, and interpretation.

## Conflicts of interest

None declared.

## Funding

Seongjoon Park is funded by JSPS (15K19517) and Shimokawa Isao is funded by JSPS (22390042).

## Supporting information


**Fig. S1** NPY deficiency does not affect mRNA levels of cardiac injury/stress genes in heart from same mice.
**Fig. S2** mRNA expression level of inflammatory cytokines is negatively correlated with WAT mass in NPY^−/−^ CR mice.
**Fig. S3** NPY and ACM inhibit isoproterenol‐induced FFA release in 3T3‐L1 adipocytes.
**Fig. S4** NPY deficiency induces WAT remodeling through Adrb3 signaling.
