# Supplementary figures and images for: Neuropeptide Y resists excess loss of fat by lipolysis in calorie‐restricted mice: a trait potential for the life‐extending effect of calorie restriction
Source: Aging Cell. 2017 Jan 19;16(2):339–48. doi: 10.1111/acel.12558 (PMC5334538; doi:10.1111/acel.12558)

Figure S1.

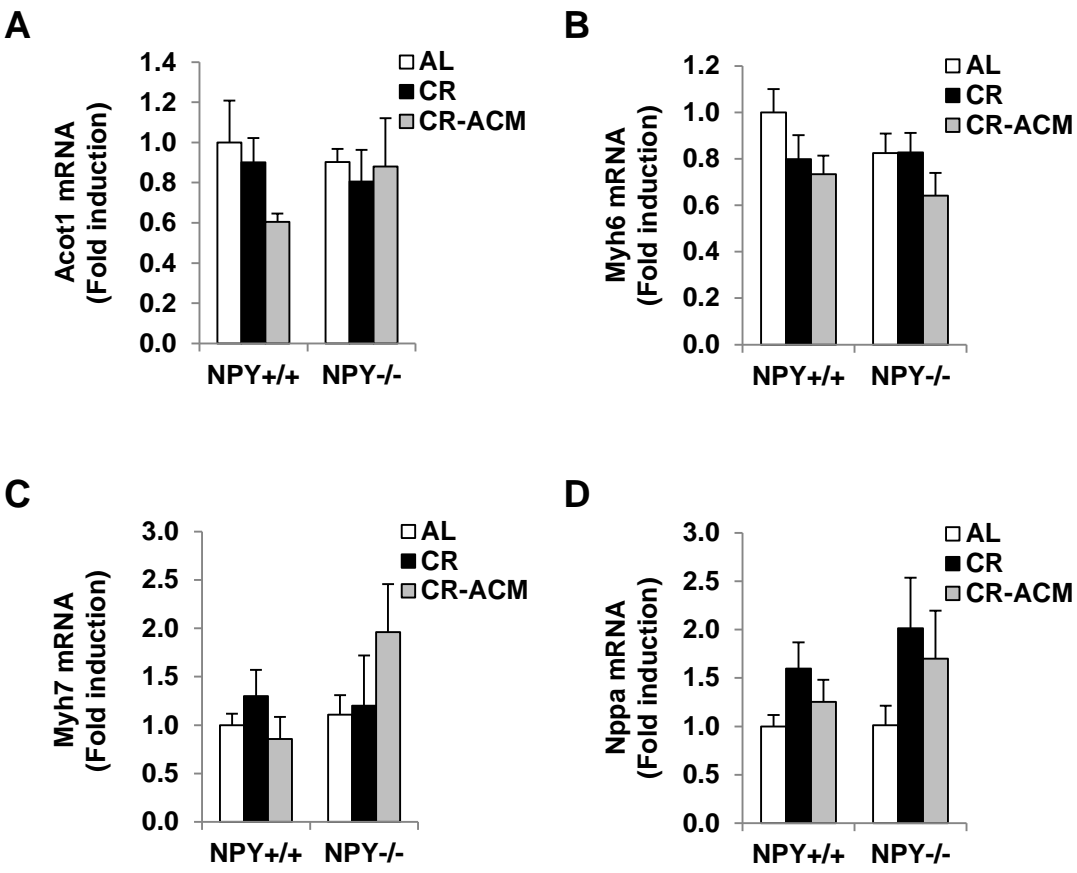

Figure S2.

A

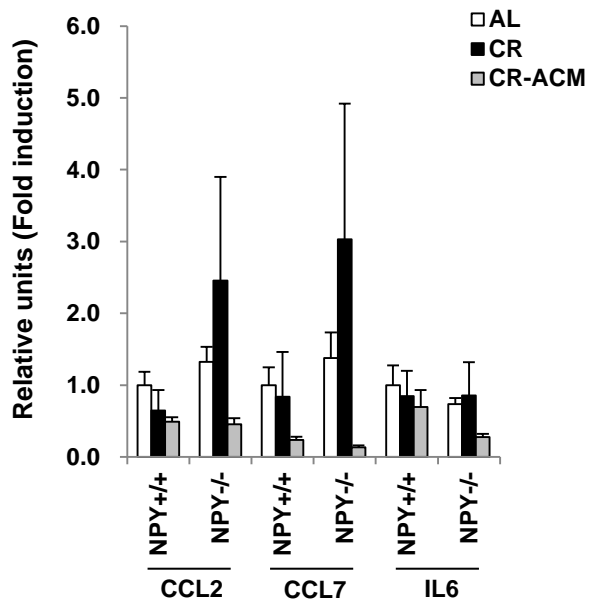

B

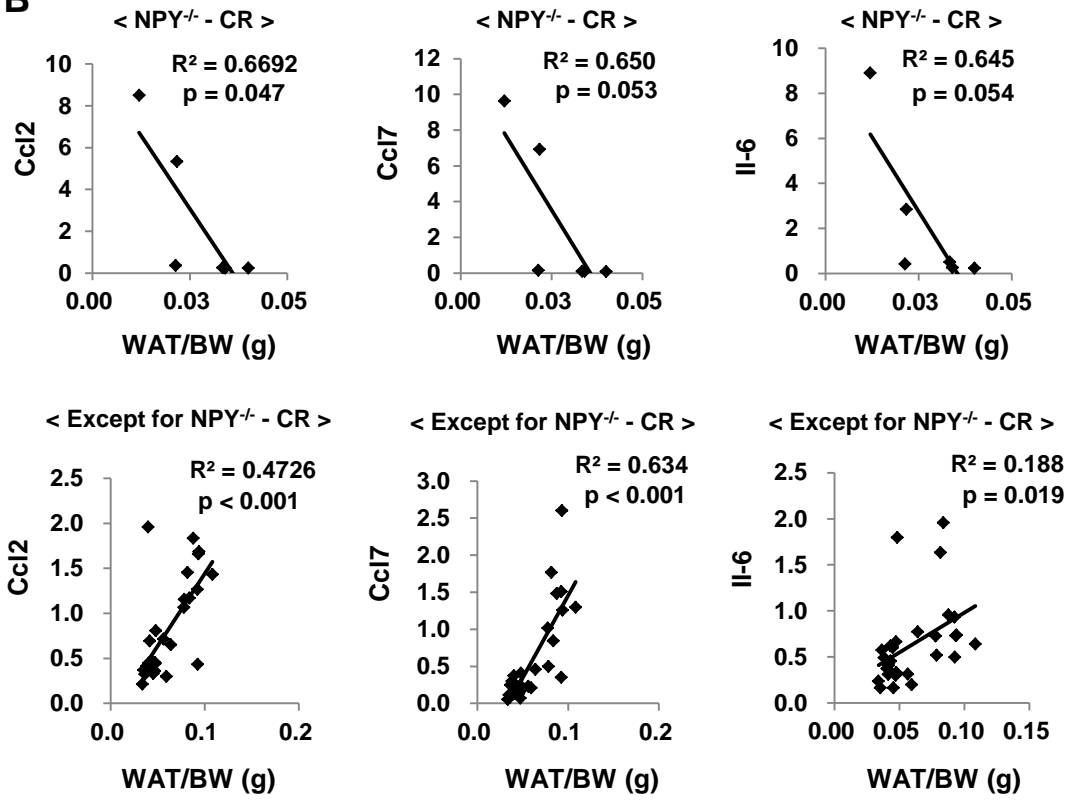

Figure S3.

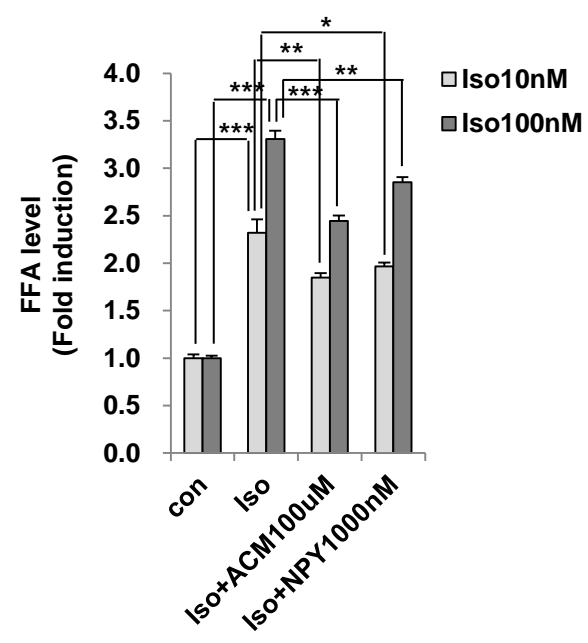

Figure S4.

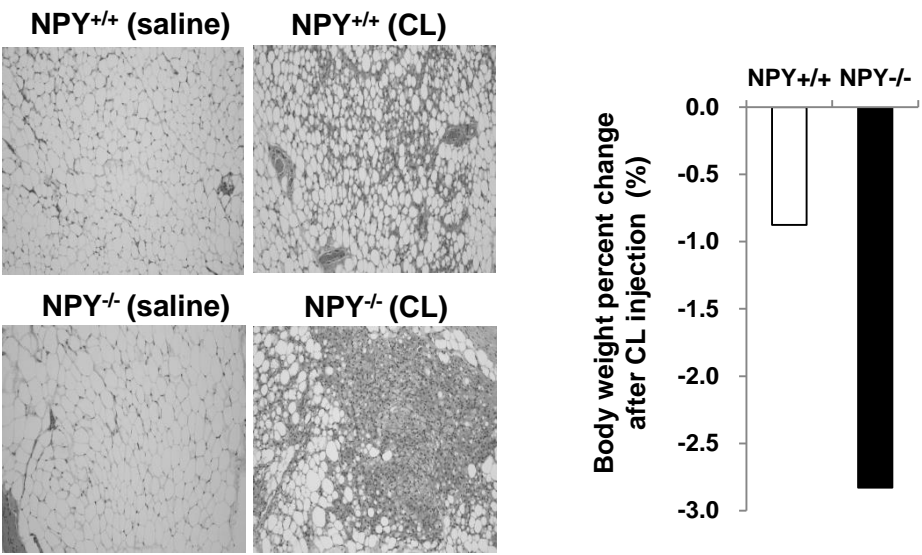

Supplement: Supplementary file 1 — Fig. S1 NPY deficiency does not affect mRNA levels of cardiac injury/stress genes in heart from same mice. Fig. S2 mRNA expression level of inflammatory cytokines is negatively correlated with WAT mass in NPY−/− CR mice. Fig. S3 NPY and ACM inhibit isoproterenol‐induced FFA release in 3T3‐L1 adipocytes. Fig. S4 NPY deficiency induces WAT remodeling through Adrb3 signaling. [file ACEL-16-339-s001.pdf]
