# Supplementary material for: Neuropeptide Y resists excess loss of fat by lipolysis in calorie‐restricted mice: a trait potential for the life‐extending effect of calorie restriction
Source: Aging Cell. 2017 Jan 19;16(2):339–48. doi: 10.1111/acel.12558 (PMC5334538; doi:10.1111/acel.12558)
Supplement: Supplementary file 2 [file ACEL-16-339-s002.docx]

**Supplementary Methods**

**Animals**

At 8 weeks of age, NPY^+/+^ and NPY^-/-^ mice were divided into AL and CR groups. The CR groups received a food allotment consisting of 70% of the mean daily food intake of the AL groups of male NPY^+/+^ and NPY^-/-^ mice every day, 30 min before lights were turned off. Mice were injected with CL-316,243 (0.1mg/kg BW) or SR59230A (5mg/kg BW) everyday for indicated days and sacrificed, and tissues were immediately collected.

**Cell culture and induction of adipocyte differentiation**

3T3-L1 cells, mice preadipocytes, were cultured in Dulbecco's Modified Eagle's medium (DMEM) containing high glucose (25 mM) and supplemented with 10% calf serum. Differentiation was induced 2 days after confluence (day 0) by adding an induction cocktail containing 100 nM insulin, 1 uM dexamethasone, and 0.5 mM 1-methyl-3-isobutyl-xanthine in DMEM/F12 medium containing 10% fetal bovine serum (FBS) for 3 days. On day 3, differentiation medium was replaced with 10% FBS DMEM/F12 medium containing with 100 nM insulin for additional 4 days, and then media were changed every 2 days. All media contained 100 U/ml penicillin, and 100 μg/ml streptomycin. All cells were maintained and differentiated at 37°C in a humidified 5% CO_2_.

**FFA quantification**

FFA level in media was measured with commercially available assay kit (WAKO pure chemical industries, Ltd., Osaka, Japan).

**Figure Legends of supplementary figure**

Supplementary figure 1. NPY deficiency does not affect mRNA levels of cardiac injury/stress genes in heart from same mice. (A-D) mRNA expression of cardiac injury/stress genes were measured by qRT-PCR in heart from same mice. All data are presented as the mean ± SEM, n = 6 per group

Supplementary figure 2. mRNA expression level of inflammatory cytokines is negatively correlated with WAT mass in NPY^-/-^ CR mice. (A) mRNA expression of inflammatory cytokines (B) Correlation of *Ccl2*, *Ccl7*, and *IL-6* mRNA levels in iWAT with WAT/BW ratio. All data are presented as the mean ± SEM; n = 5-6 per group.

Supplementary figure 3. NPY and ACM inhibit isoproterenol-induced FFA release in 3T3-L1 adipocytes. At day 7, 100uM of ACM and 1uM of NPY were pretreated for 5 min then treated 10nM or 100nM of isoproterenol (Iso) for 1h. All data are presented as the mean ± SEM; ^*^p < 0.05, ^**^p < 0.01, and ^***^p < 0.001.

Supplementary figure 4. NPY deficiency induces WAT remodeling through Adrb3 signaling. HE staining of iWAT from NPY^+/+^ and NPY^-/-^ mice injected with CL-316,243 (CL, 0.1mg/kg BW) every day for 1 week and body weight percent change after injection
